# How a Replication Origin and Matrix Attachment Region Accelerate Gene Amplification under Replication Stress in Mammalian Cells

**DOI:** 10.1371/journal.pone.0103439

**Published:** 2014-07-25

**Authors:** Shun-suke Tanaka, Sho-hei Mitsuda, Noriaki Shimizu

**Affiliations:** Graduate School of Biosphere Science, Hiroshima University, Higashi-hiroshima, Hiroshima, Japan; Helmholtz Zentrum München, Germany

## Abstract

The gene amplification plays a critical role in the malignant transformation of mammalian cells. The most widespread method for amplifying a target gene in cell culture is the use of methotrexate (Mtx) treatment to amplify dihydrofolate reductase (*Dhfr*). Whereas, we found that a plasmid bearing both a mammalian origin of replication (initiation region; IR) and a matrix attachment region (MAR) was spontaneously amplified in mammalian cells. In this study, we attempted to uncover the underlying mechanism by which the IR/MAR sequence might accelerate Mtx induced *Dhfr* amplification. The plasmid containing the IR/MAR was extrachromosomally amplified, and then integrated at multiple chromosomal locations within individual cells, increasing the likelihood that the plasmid might be inserted into a chromosomal environment that permits high expression and further amplification. Efficient amplification of this plasmid alleviated the genotoxicity of Mtx. Clone-based cytogenetic and sequence analysis revealed that the plasmid was amplified in a chromosomal context by breakage-fusion-bridge cycles operating either at the plasmid repeat or at the flanking fragile site activated by Mtx. This mechanism explains how a circular molecule bearing IR/MAR sequences of chromosomal origin might be amplified under replication stress, and also provides insight into gene amplification in human cancer.

## Introduction

Gene amplification is a topic of central interest in the fields of genome evolution, malignant transformation, and industrial protein production. Increase in the gene copy number allow the copies to diverge, a process that is critical for the creation of novel genes over the course of evolution. Amplification of oncogenes and drug-resistance genes has been observed in many human cancers, and this phenomenon is closely associated with the establishment of the malignant state via overproduction of specific protein products (reviewed in [1], [2]). Highly amplified genes in human cancer cells reside on two types of cytogenetic structures, namely, extrachromosomal double minutes (DMs) and chromosomal homogeneously staining regions (HSRs). The gene amplification in most of the cases leads to the overproduction of corresponding proteins In the context of cancer, amplification confers a growth advantage, whereas in the context of industrial protein production, amplification of genes has been widely used to obtain higher yields of various recombinant protein pharmaceuticals [3].

To investigate gene amplification, it is necessary to use an *in vitro* method for inducing amplification of a target gene in cultured cells. The most widely used method is amplification of a plasmid bearing dihydrofolate reductase (*Dhfr*) gene by treatment with methotrexate (Mtx), a specific inhibitor of DHFR protein, in *Dhfr*-deficient Chinese hamster ovary (CHO) cells such as strain DG44 [3]. This method produces chromosomal HSRs, but rarely produces DMs. The major drawbacks of this method are the large amounts of time [4], labor, and experience required; furthermore, the amplified structures are frequently unstable over prolonged culture periods [5].

**Figure 1:**
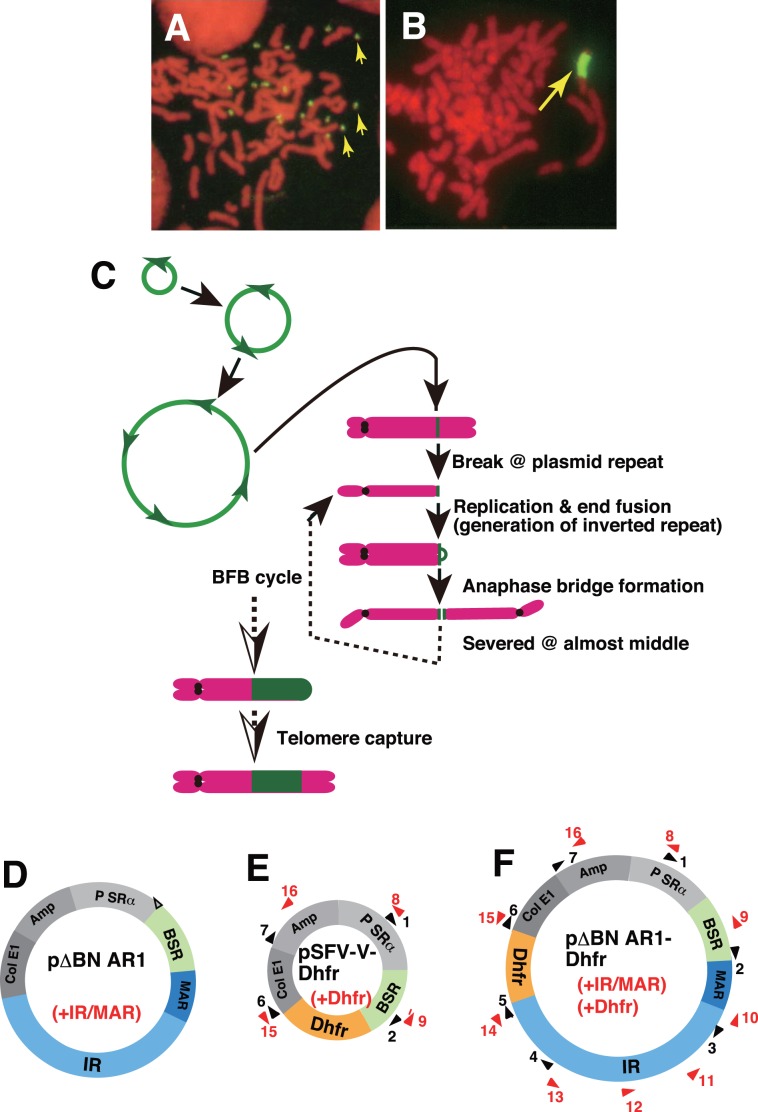
Amplification of the initiation region/matrix attachment region (IR/MAR) plasmid. The IR/MAR (+) plasmid generated double minutes (DMs) (A) or homogeneously staining regions (HSRs) (B) in human COLO 320 cells. In these images, plasmid sequence was detected by FISH (green; indicated by arrows), and chromosomes were counterstained with propidium iodide (PI; red). A previous study revealed the mechanism by which the IR/MAR plasmid generates DMs or HSRs in these cells; these findings are summarized in (C). In the drawing, the plasmid sequence is green, the chromatid is orange, and the centromeres are black circles. In (D)–(F), structures of the three plasmids used in this study are shown. The orientation and position of each PCR primer used for the detection of inverted repeats are depicted as black and red triangles with accompanying numbers. Black and red indicate hybridization to different DNA strands.

On the other hand, we previously showed that a plasmid with a mammalian replication initiation region (IR) and a nuclear matrix attachment region (MAR) was spontaneously, and very efficiently, amplified in transfected cells [6], [7]. This amplification, which was dependent upon the cell type used, spontaneously generated many DMs (Fig. 1A), as well as long and homogenous HSRs composed solely of plasmid repeats (Fig. 1B), in human colorectal carcinoma COLO 320 cells. By contrast, in hamster CHO DG44 cells, the IR/MAR plasmid generated chromosomal paired-dot or multiple-dot signals along the chromosome arm, a pattern we called “fine-ladder HSR” [8].

In previous work, we revealed the mechanism of IR/MAR plasmid amplification in COLO 320 cells, shown schematically in Figure 1C. Initially, the IR/MAR plasmid is maintained and multimerized at an extrachromosomal site [6], [9], [10]. These extrachromosomal molecules can be detected by fluorescence in situ hybridization (FISH), and they appear as DMs if multimerization proceeded extensively. In these molecules, the plasmid sequences are arranged as highly ordered tandem repeats [6]. For amplification to occur, sequence information from both IR and MAR is required; unrelated sequence of similar length does not support amplification [11]. The minimum sequence required for amplification overlaps with the sequence required for replication initiation in a chromosomal context [10], [11]; thus the IR/MAR sequence is most likely required for extrachromosomal maintenance and multimerization at least during the initial stage after the transfection. If the extrachromosomal multimer suffers from DNA damage, it is either eliminated from the cells [12] or integrated into the chromosome arm. In the latter context, the multimer initiates a breakage-fusion-bridge (BFB) cycle that generates large HSRs [6] (Fig. 1A). This cycle involves breakage at the plasmid repeat, followed by replication/end fusion resulting in generation of a dicentric chromosome and chromatin bridge during the anaphase. Breakage of the bridge at a point shifted from the exact middle resulted in unequal segregation of the plasmid to daughter cells; subsequently, cells with longer HSRs could be selected under pressure. The model was consistent with several observations. First, the HSR was usually located at the end of a metaphase chromosome, and it generated an anaphase chromatin bridge with the plasmid repeat located at the middle [6]. In these cells, the bridge was usually broken near its center [9]. Many inverted recombinations occurred among amplified plasmid sequences generated by replication/end fusions [13]. The BFB cycle might be terminated by telomere capture [9].

The IR/MAR and the *Dhfr*/Mtx gene amplification methods are based on quite different rationales. However, we recently found that the presence of IR/MAR sequence in a *Dhfr* gene-bearing plasmid greatly accelerated *Dhfr*/Mtx amplification in CHO DG44 cells; furthermore, it generated far more stable structures in the absence of Mtx (or in the presence of low concentrations of this drug) [14]. This method, which we called “IR/MAR-Dhfr fusion”, provides an advanced method for producing stable cloned cells that produce higher amounts of recombinant protein. Furthermore, as discussed below, several lines of evidence suggest that the IR/MAR-*Dhfr* fusion method experimentally mimics oncogene amplification in cancer cells. In this study, we sought to determine how the IR/MAR sequence accelerates *Dhfr*/Mtx amplification.

## Materials and Methods

### Plasmids

Plasmids pΔBN.AR1 [6], pSFV-V-Dhfr [14] and pΔBN AR1-Dhfr. [14] were constructed by us in previous studies; their structures are outlined in Figures 1D–F. pΔBN AR1 and pΔBN AR1-Dhfr contained a 4.6 kb IR fragment including Ori β derived from the region 3′-downstream of *Dhfr* gene and a MAR sequence from the Ig κ intron. pSFV-V-Dhfr and pΔBN AR1-Dhfr contained an expression cassette for the *Dhfr* gene. All three plasmids contained a blasticidine resistance gene (*BSR*). Plasmid DNA was purified using the PureLink HiPure Plasmid Midiprep kit (Life Technologies Inc.).

### Cell culture

CHO DG44 cells, obtained from Dr. Lawrence Chasin at Columbia University, were maintained in α-MEM medium with nucleotide (Sigma, M8042) supplemented with 10% fetal bovine serum (FBS), as previously described [8], [14]. Plasmid DNA was transfected into cells using Lipofectamine 2000 reagent (Invitrogen) and Opti-MEM (Gibco). One day after transfection, 5 µg/ml of blasticidine was added to select for transformants, and the concentration was increased to 10 µg/ml on day 10. Usually, on day 23, the medium was replaced with α-MEM without nucleotide (Sigma, M4526) supplemented with 10% dialyzed FBS (Thermo Scientific). Methotrexate (Sigma) was dissolved in water and added to the culture at 5 or 50 nM. Cloning of cells was performed by limiting dilution.

### Cytochemical procedures

FISH using metaphase chromosome spreads, a DIG-labeled probe prepared from pΔBN AR1-Dhfr plasmid DNA, and FITC-conjugated mouse anti-digoxigenin (DIG) antibody (Molecular probe) was performed as described previously [6]. For FISH of paraformaldehyde (PFA)-fixed samples, cells were grown on cover glass, fixed with 4% PFA in phosphate buffered saline without divalent cation (PBS-) for 10 min at room temperature, permeabilized with 0.5% NP-40 in PBS- for 10 min at room temperature, washed with PBS-, and treated with methanol at −20°C for 3 min. Fixed cells were stored in 70% ethanol at 4°C, re-hydrated with PBS-, and hybridized with the probe, as described.

### Detection of inverted repeats by PCR

Genomic DNA was prepared from cells according to a standard protocol. To detect the inverted repeat, we used a series of primer sets that were arranged in the same orientation along the plasmid sequence, as described previously [13]. The positions and orientations are indicated in Figures 1D–F, and their names and sequences appear in Table S1. The reaction mixture (20 µl) contained 1× Blend Taq Buffer (Toyobo), 200 µM dNTP, 0.12 units of Blend Taq plus polymerase (TOYOBO), 15 ng genome DNA, and 1 µM of each primer. PCR conditions were 35 cycles of 94°C for 30 sec, 60°C for 30 sec, and 72°C for 3 min.

### Chromatin immunoprecipitation (ChIP)

Preparation of chromatin, immunoprecipitation, isolation of DNA, and PCR analysis were performed by standard protocols. Briefly, cells were fixed with 1% PFA for 5 min at room temperature, treated with 0.5% NP-40, collected, and lysed with 1% sodium dodecyl sulfate (SDS). Chromatin was sheared using a sonicator (Active Motif Co.), and immunoprecipitation was performed with mouse monoclonal anti-histone H3K9-trimethyl (H3K9me3) antibody (Active Motif Co.), anti-histone H3K9-acetyl (H3K9ac) antibody (Active Motif Co.), mouse(G3A1)monoclonal IgG1 isotype control (Cell Signaling Technology Inc.), and protein G magnetic beads (Active Motif). DNA was purified and subjected to real-time PCR analysis using Thunderbird SYBR qPCR Mix (Toyobo), the Step One Real time PCR System (Applied Biosystems), and a primer set that amplified a 125 bp segment of the *Dhfr* coding sequence.

## Results

### IR/MAR increases transformation efficiency, initial extrachromosomal multimerization, and later chromosomal amplification

**Figure 2:**
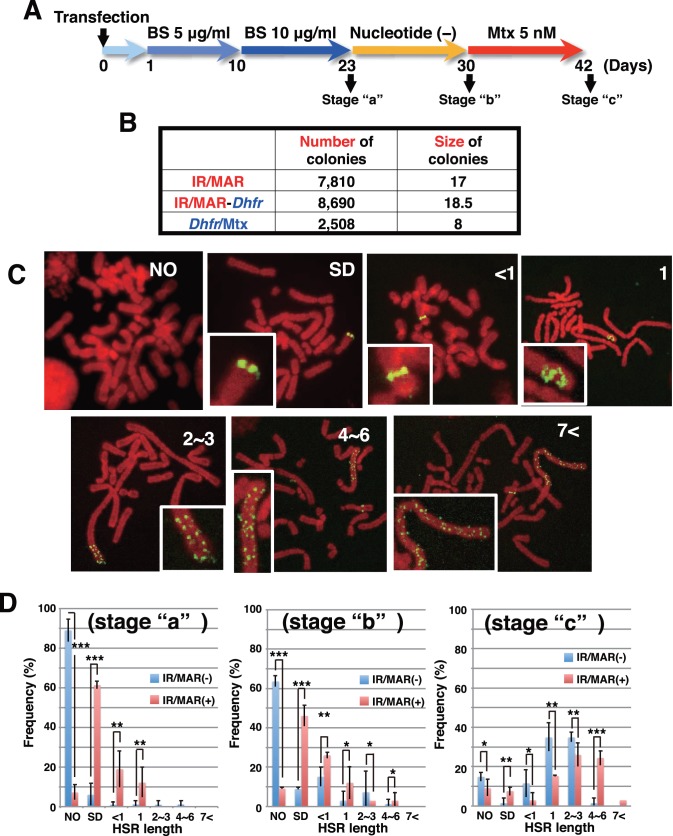
The initiation region/matrix attachment region (IR/MAR) sequence increased transformation efficiency, initial multimerization, and chromosomal extension. Three plasmids, shown in Figures 1D–F, were transfected to 1.5×10^6^ CHO DG44 cells. The selection procedure is shown in (A). After selection with BS (stage “a”), total colony number from 8×10^5^ tranfected cells and colony size in arbitrary units were scored at the stage “a” (B). At each selection stage, metaphase chromosome spreads were prepared, and plasmid sequences were detected by FISH. (C) Representative images for no signal (NO), paired single dots (SD), and homogeneously staining regions (HSRs) of various sizes (<1 to >7, expressed in units of chromosome width. (D) The frequency of each amplification status among cells receiving pSFV-V-Dhfr (dihydroflate reductase) (IR/MAR−) or pΔBN AR1-Dhfr (IR/MAR+) at each step was scored by examining 30–40 metaphases in triplicate. Values represent means; error bars indicate +/− standard deviation. ***; p<0.001, **; p<0.01, *; p<0.1.

To investigate the effect of the IR/MAR sequence on *Dhfr*/Mtx amplification in CHO DG44 cells, we transfected these cells with three plasmids (Figs. 1D–F) and selected transformants with blasticidin (BS; stage “a” of Fig. 2), followed by nucleotide-deficient medium (stage “b” of Fig. 2) and 5 nM Mtx in the same medium (stage “c” of Fig. 2). Upon examination of the transformation efficiency at stage “a”, we found that the presence of the IR/MAR sequence in the plasmid resulted in more, and larger, colonies (Fig. 2B), suggesting that IR/MAR promoted higher BSR expression at the initial stage of transformation. Because expression from an extrachromosomal context is usually high, this phenomenon most likely reflects the extrachromosomal maintenance of the IR/MAR plasmid at the initial stage.

At stages “a”, “b”, and “c”, we prepared metaphase chromosome spreads and analyzed them by FISH using a plasmid-derived probe. Representative images are shown in Figure 2C. Individual metaphases contained no signal (NO), paired single dots (SD), or HSRs of various lengths. Among transformants containing pSFV-V-Dhfr (IR/MAR−) or pΔBN AR1-Dhfr (IR/MAR+) at each stage, we determined the frequencies of cells bearing HSRs of various sizes (Fig. 2D). At stage “a”, most transformants with the IR/MAR (−) plasmid did not contain a plasmid signal, whereas cells with the IR/MAR (+) plasmid contained an amplified plasmid sequence as an SD or longer HSR. According to our previous study, we considered SD signals to reflect extrachromosomal multimerization followed by integration into the chromosome arm [6], [10]. Furthermore, at stages “b” and “c”, HSRs were much more elongated in the IR/MAR (+) population relative to the IR/MAR (−) population. Consistent to our previous study [14], these data clearly showed that the IR/MAR (+) plasmid was spontaneously amplified after simple BS selection, and that it was more efficiently elongated in a chromosomal context in nucleotide-deficient medium containing a low concentration of Mtx.

### IR/MAR increases the number of HSRs per cell

**Figure 3:**
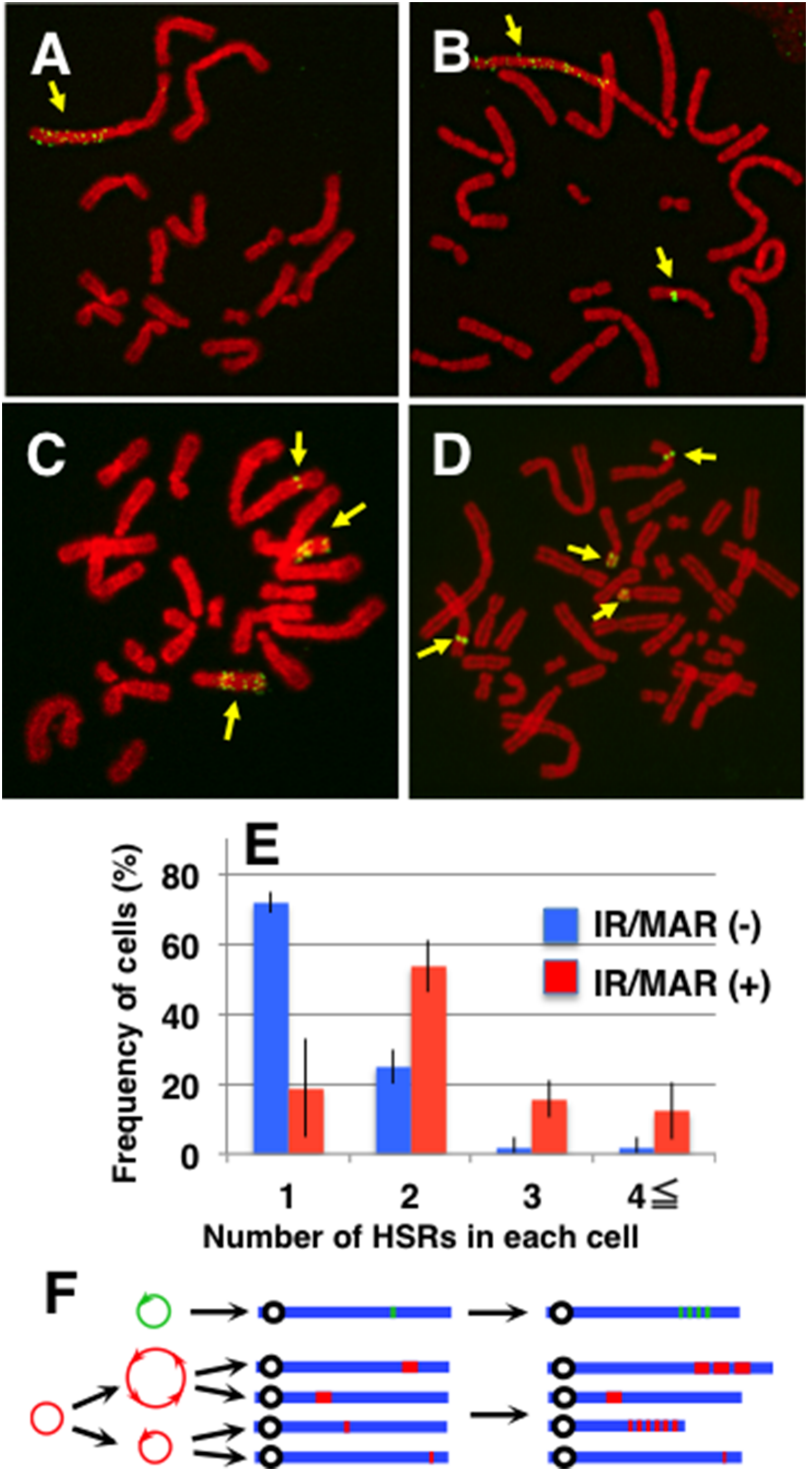
The initiation region/matrix attachment region (IR/MAR) increased the number of homogeneously staining regions (HSRs) per cell. At stage “c” in Figure 2A, metaphase spreads were prepared from the IR/MAR (−) and (+) cultures, and these were analyzed by FISH. Representative images are shown in (A)–(D); HSRs are indicated by yellow arrows. The number of HSRs per cell was determined by examining 19–22 metaphases, which had at least one HSR, in three replicates, and means +/− standard deviation are plotted in (E). The result from Figures 2 and 3 are summarized in panel F, in which green and red represent IR/MAR (−) and (+) plasmids respectively, blue represents chromatin, and black circles represent centromeres.

During the experiments described above, we noticed that the number of HSRs per cell was much higher in IR/MAR (+) than in IR/MAR (−) cultures. Representative metaphase images are shown in Figures 3A–D, in which one (A), two (B), three (C), or four (D) HSRs (pointed by arrows) appear in cells. Importantly, the length of individual HSRs varied significantly within a cell, suggesting that HSR elongation depends heavily on chromosomal context. We counted the number of HSRs per cell at stage “c” (Fig. 2A), and plotted the frequency (Fig. 3E). The IR/MAR (−) plasmid generated predominantly single HSRs (43 cells among 60 metaphase cells), whereas the IR/MAR (+) plasmid generated multiple HSRs per cell (47 cells among 58 metaphase cells). The average number of HSR per cells was 1.33 and 2.36 for IR/MAR (−) and (+), respectively. This is our novel finding in this study. The results from Figures 2 and 3 are schematically summarized in Figure 3F: the IR/MAR (+) plasmid was multimerized in an extrachromosomal context and integrated at multiple chromosomal sites, where the HSR was additionally elongated, or not, depending on the chromosomal context.

### IR/MAR increases the apparent HSR elongation rate and decreases the frequency of micronuclei after the increase in the Mtx concentration

**Figure 4:**
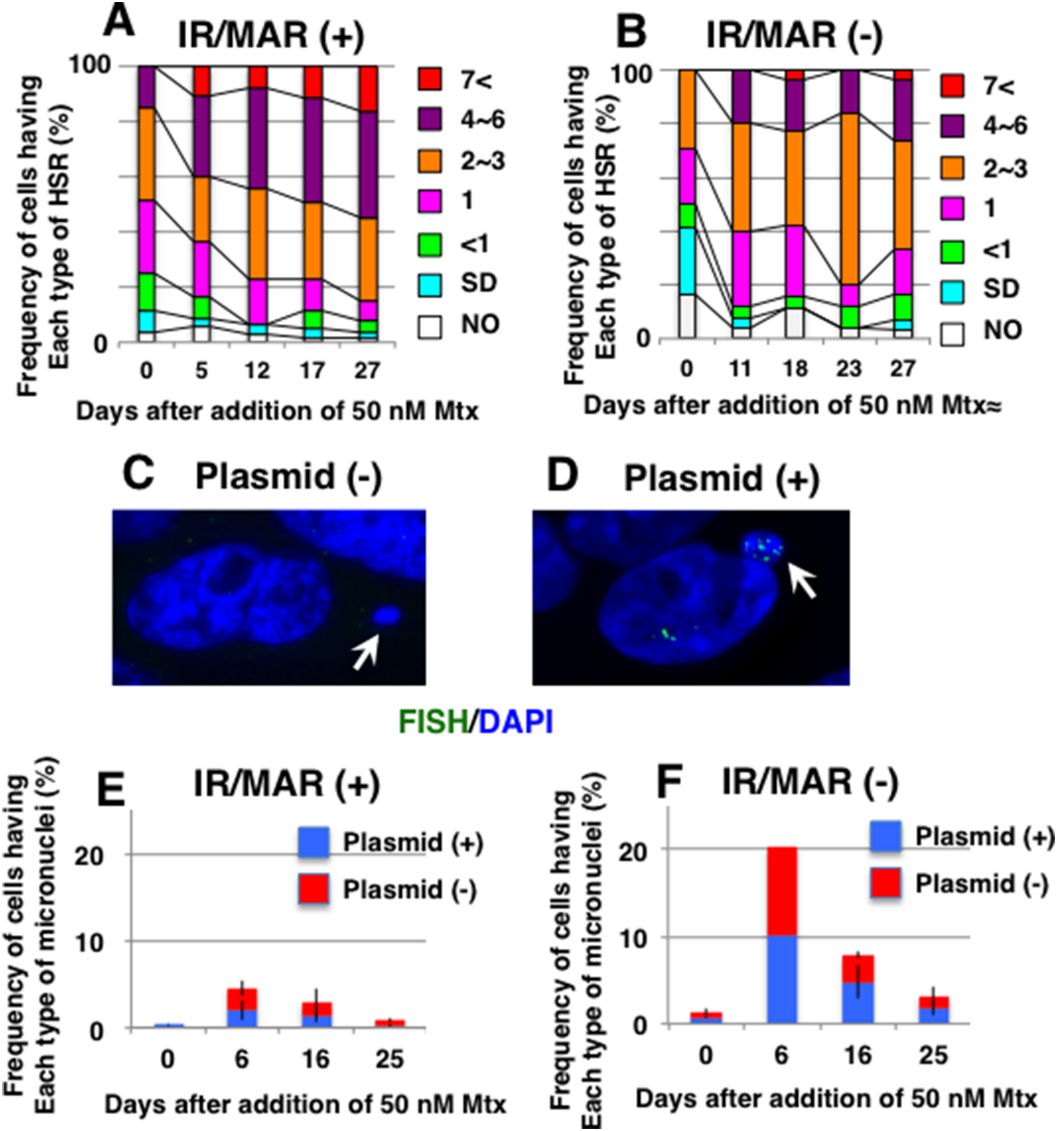
The initiation region/matrix attachment region (IR/MAR) increased the homogeneously staining region (HSR) elongation rate and decreased the frequency of micronuclei after elevation of methotrexate (Mtx) concentration. The IR/MAR-bearing plasmid (A, E) or a plasmid lacking this sequence (B, F) was transfected into Chinese hamster ovary DG44 cells. Cells growing in 5 nM Mtx (stage “c” in Fig. 2A) were subsequently cultured in 50 nM Mtx. Then, metaphase spreads were prepared at the indicated times (in days), and plasmid sequences were detected by FISH. The frequency of cells bearing HSRs of various lengths was determined by observing 30–60 metaphase cells; the results are plotted in (A) and (B). Cells without (C) and with (D) plasmid HSRs contained micronuclei (arrowed). Frequencies were scored by observation of 200–500 cells in three replicates, and means +/− standard deviation are plotted in (E) and (F).

After the growth in 5 nM Mtx (stage “c” in Fig. 2A), the increase in the Mtx concentration to 50 nM resulted in further elongation of the HSRs. This elongation, which started within 5 days after the Mtx increase and continued until day 27, was faster in IR/MAR (+) cultures (Fig. 4A) than in IR/MAR (−) cultures (Fig. 4B). The faster elongation in the presence of IR/MAR was consistent with the results of our previous study [14].

The cells also contained micronuclei, small nucleus-like structures in the cytoplasm that are induced by clastogenic or aneugenic stress. These structures either lacked (Fig. 4C) or contained (Fig. 4D) plasmid sequences. The latter suggested the incorporation of HSRs into the micronuclei. Mtx is an inhibitor of DHFR, and it causes clastogenic DNA strand breakage by lowering the cellular nucleotide pool [15], [16], which explains the induction of micronuclei as other clastogenic reagents usually induced them. When we counted the micronuclei after the Mtx increase, and plotted the results (Figs. 4E and F), the results clearly showed that the number of micronuclei were less frequent in IR/MAR (+) than in IR/MAR (−) cultures. It was the case irrespective of whether the micronuclei contained plasmid HSR or not.

This observation suggests that the IR/MAR lowered the frequency of Mtx-induced strand breakage within or outside of HSRs, possibly because DHFR protein expression was higher in IR/MAR (+) during the initial period after the Mtx elevation. This would explain why the presence of IR/MAR in the plasmid increased the stability of the amplified genes, an important merit of using the IR/MAR sequence [14].

### The BFB cycle elongates fine-ladder HSRs

**Figure 5:**
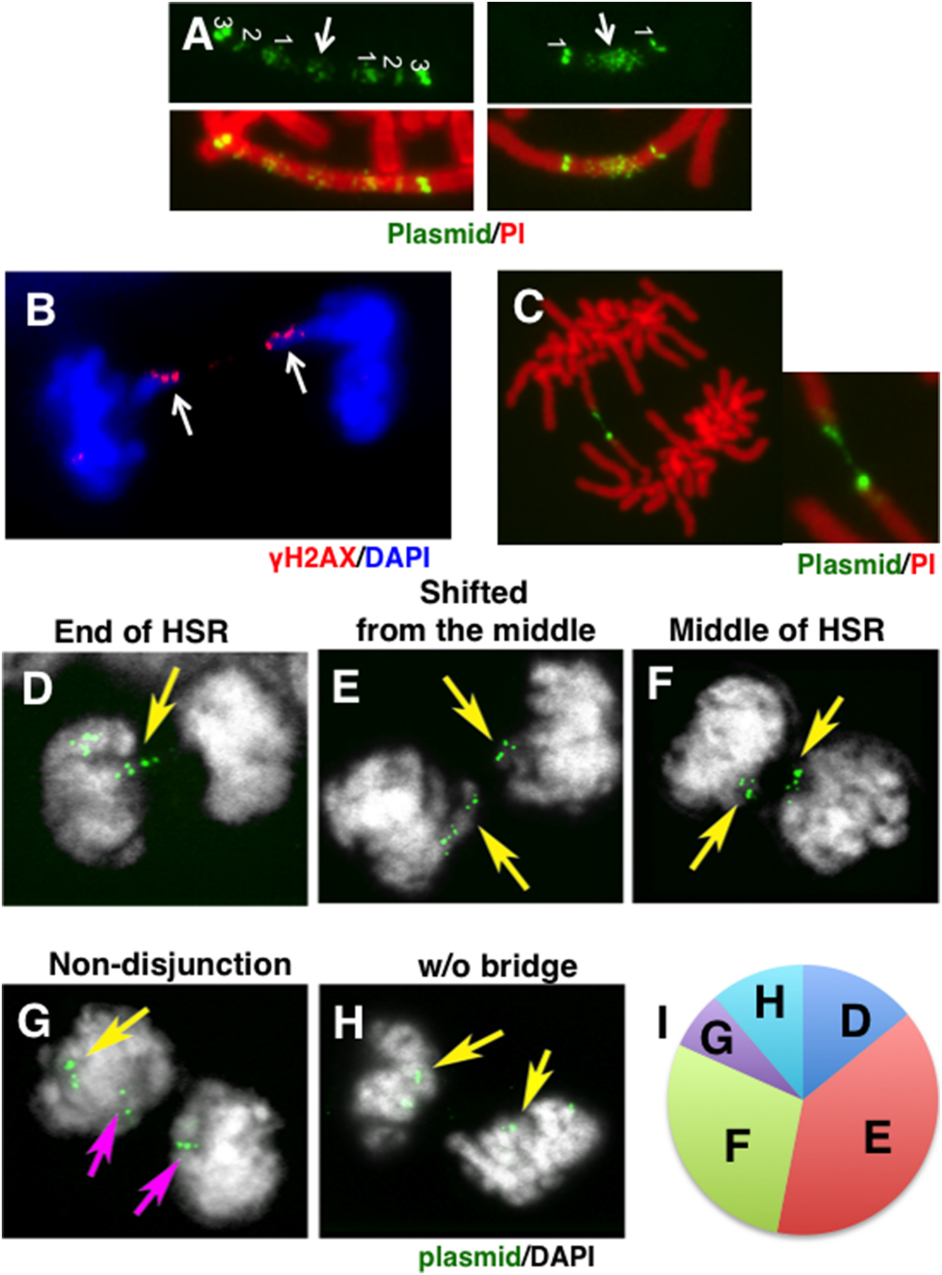
The breakage-fusion-bridge (BFB) cycle operates at chromosomal sites to generate and elongate fine-ladder homogeneously staining regions (HSRs). Metaphase spreads (A, C) or paraformaldehyde (PFA)-fixed samples (B, D–I) from cells after BS selection (panels B and C) or cells grown in the presence of 5 nM methotrexate for 12 days (A, D to I) were analyzed. Panel A shows two representative images of symmetrical plasmid signals (green) arranged along the metaphase chromosome (red). Some cells contained anaphase bridges; (B) shows γ-H2AX signal (red) at the broken ends of a bridge, and (C) shows a bridge severed in the middle. Panels (D)–(H) show representative images of the segregation of fine-ladder HSRs (green) with anaphase chromosomes (gray). A chromatin bridge composed of a fine-ladder HSR could be severed at its end (D), at a site shifted from the middle of the HSR (E), or at the middle of the HSR (F). An HSR might also be delivered to one daughter nucleus by non-disjunction (yellow arrow in G), or evenly segregated without bridge formation (H). Frequencies of these events among the culture were counted and plotted in panel (H).

As described, we previously revealed that HSRs composed of a homogeneous plasmid array are elongated in COLO 320DM cells by the BFB cycle (Fig. 1C). Here, we sought to determine how the fine-ladder HSRs are elongated in CHO DG44 cells. Consistent with the BFB model, long HSRs were frequently located at the ends of metaphase chromosomes (Figs. 2C and 3A–D). Furthermore, in some metaphase chromosomes, the plasmid repeats were arranged symmetrically along the arms (Fig. 5A), suggesting the stabilization of a dicentric chromosome generated during the BFB cycle by inactivation of a centromere. Many anaphase chromatin bridges were present, and gamma-H2AX signals, which reflect chromatin breakage, were detected at the broken ends of bridges (Fig. 5B). During BS selection, most of the bridges were severed in the middle, where the plasmids were located (Fig. 5C). Remarkably, after Mtx addition, bridges composed of HSRs were not necessarily severed at the middle, but instead were severed at the ends of the HSRs (Fig. 5D), at sites shifted from the middle (Fig. 5E), or in the middles of HSRs (Fig. 5F). In addition, in some anaphase cells, an HSR was delivered to one daughter cell by non-disjunction (Fig. 5G), whereas in other cells, HSRs were evenly segregated without a bridge, possibly reflecting stabilization by telomere capture (Fig. 5H). The frequency of these events is shown in Figure 5I. These observations suggested that the BFB cycle was operating in these cells. However, unlike elongation in COLO 320 cells, Mtx-induced elongation did not involve breakage at the plasmid repeat, but instead involved breakage at a chromosome site adjacent to the plasmid repeat, most likely the chromosomal fragile site activated by Mtx. These data were consistent with the published papers showing that Mtx activated fragile site and initiated the BFB cycle [17]–[19].

### Detection of the junction of inverted recombination in cloned cells suggests a mechanism by which Mtx elongates or shortens HSRs

We next clonally isolated cells from cultures selected with BS, i.e., step “a” in Figure 2A. These clones were first analyzed by FISH to evaluate plasmid amplification, and representative clones were selected. Eight and four clones were selected from the IR/MAR (+) and (−) cultures, respectively, and these clones were cultured in nucleotide-deficient medium for 5–9 days, followed by culture in 5 nM Mtx for 23–39 days. After again detecting the plasmid sequence by FISH, we classified these clones into four categories according to their patterns of plasmid amplification before and after Mtx treatment. Genomic DNA was isolated from these cells, and subjected to PCR using specific primer sets (Table S1). These primer sets annealed to the same strand of the plasmid (Figs. 1E and F), and amplified product only from junctions of inverted recombinations [13]. Therefore, the genomic DNA from non-transfected CHO DG44 cells or the plasmid DNA did not produce any PCR band under these conditions (not shown).

**Figure 6:**
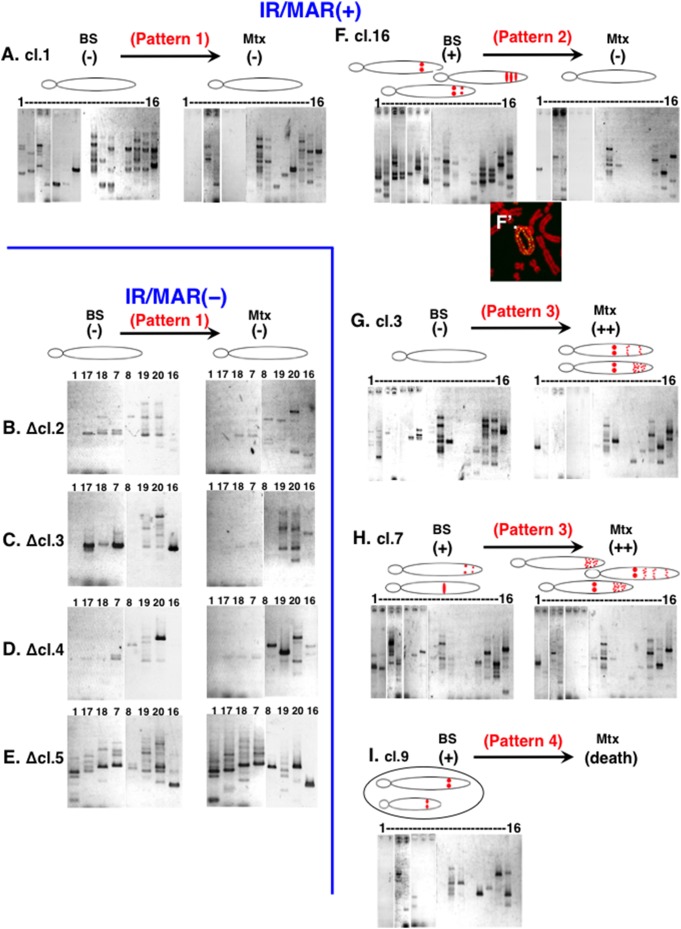
Clonal analysis of homogeneously staining region (HSR) elongation and inverted recombination. After selection with blasticidine (BS) (stage “a” in Fig. 2A), cells were cloned by limiting dilution. Each clone was cultured in nucleotide-deficient medium containing 5 nM methotrexate (Mtx). The gene amplification status revealed by FISH before and after Mtx treatment is drawn schematically in each panel; plasmid signals are depicted in red. The inverted repeat in the plasmid sequence was detected by PCR, and images of ethidium bromide-stained agarose gels are shown in each panel. The numbers above each gel image correspond to the primer set used (Table S1). The results obtained with the initiation region/matrix attachment region (IR/MAR) (+) plasmid are shown in panels (A), (F)–(I), and results obtained with the IR/MAR (−) plasmid are shown in panels (B)–(E).

Pattern 1 was the case in which the FISH signal was barely detectable both before and after Mtx treatment (Fig. 6A). Representative clone 1, which was obtained from the IR/MAR (+) plasmid, yielded many PCR bands representing inverted recombination in the plasmid repeat. In a previous paper, we showed that such inverted recombination is specifically detected in HSRs but not in DMs [13]. Remarkably, the number of bands did not differ significantly before and after Mtx treatment. Because clone 1 did not exhibit plasmid amplification after Mtx treatment, we conclude that the BFB cycle at plasmid repeats did not contribute to elongation of HSRs. On the other hand, all four clones obtained from the IR/MAR (−) plasmid also belonged to pattern 1. Importantly, these cells also contained many inverted recombinations (Figs. 6B–E), suggesting that such recombination might occur after integration of the transfected plasmid into the chromosome arm, irrespective of the presence or absence of IR/MAR.

Pattern 2 was the case in which the cells had an HSR before Mtx treatment, but did not have one afterward. Representative clone 16 (Fig. 6F) had many inverted recombinations before Mtx, but lost most of them after the treatment. Consistent with this, this clone contained a ring chromatid comprising a fine-ladder HSR (Fig. 6F, bottom); thus deletion of an HSR by (e.g.,) intra-allelic recombination between the plasmid repeats might explain pattern 2.

**Figure 7:**
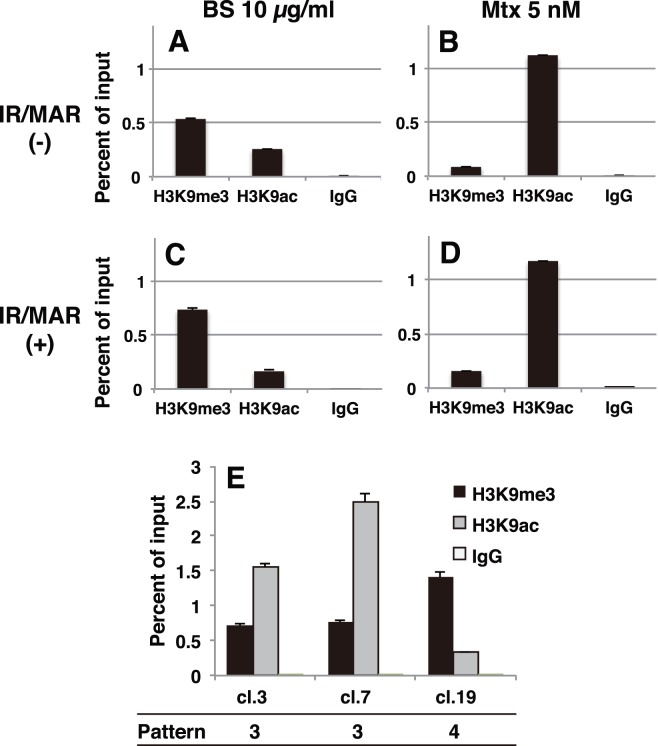
H3K9 modification before and after the methotrexate (Mtx) treatment. CHO DG44 cells were transfected with initiation region/matrix attachment region (IR/MAR) (−) pSFV-V-Dhfr (dihydroflate reductase) or IR/MAR (+) pΔBN AR1-Dhfr, as before. Chromatin was isolated from polyclonal transformants at stages “a” and “c” in Figure 2A (A–D). Alternatively, prior to Mtx treatment, chromatin was isolated from indicated clones classified as pattern 3 or 4. The chromatin was immunoprecipitated using anti-histone H3K9-trimethyl (H3K9me3) or anti-histone H3K9-acetyl (H3K9ac) antibody, and the precipitate was analyzed by real-time PCR.

Pattern 3 was the case in which the HSR was elongated after Mtx treatment. Clone 3 (Fig. 6G) and 7 (Fig. 6H) belonged to this pattern. In both cases, the PCR analysis revealed that the number of bands did not differ significantly during Mtx treatment. As in the case of pattern 1, this observation suggested that inverted recombination (i.e., the BFB cycle at the plasmid repeat), did not elongate the fine-ladder HSR under these conditions.

Pattern 4 was the case in which cells died during Mtx treatment. Figure 6I shows representative clone 9. These cells died because they could not express enough DHFR protein, which was required for the survival in the presence of Mtx, possibly because the chromosomal integration site of the plasmid was not adequate for sufficient expression.

These data suggested that the inverted repeats were spontaneously generated after the insertion of the plasmid repeat to the chromosome arm irrespective of the presence or absence of the IR/MAR. On the other hand after the addition of Mtx, the generation of the fine ladder HSR was not accompanied by the additional inverted repeat in the plasmid sequence. Furthermore, the fine ladder HSR might be excised from the chromosome arm during the Mtx treatment. These findings will be discussed in order to develop a model.

### Histone H3K9 is methylated before Mtx treatment, but acetylated in cells growing in Mtx

We next examined the modification of histone H3K9, because it is tightly related to the chromatin relaxation state that determines transcription and recombination. We isolated chromatin from the cells growing in BS (stage “a” of Fig. 2A) and the cells after Mtx treatment (stage “c” of Fig. 2A), and precipitated this chromatin using modification-specific antibodies against H3K9. In both the IR/MAR (+) and (−) plasmid cultures, chromatin from cells growing in BS was more trimethylated than acetylated at histone H3K9 (Fig. 7A, C). On the other hand, Mtx treatment greatly increased acetylation and decreased methylation at H3K9 (Fig. 7B, D).

We next investigated the clones analyzed in Figure 6. Pattern 3 clones (clones 3 and 7), in which amplification proceeded in response to Mtx, had more acetylated H3K9 than methylated H3K9 prior to Mtx treatment (Fig. 7E). By contrast, the pattern 4 clone (clone 19), which died after Mtx treatment, had more methylation than acetylation on H3K9 (Fig. 7E). These results suggested that most of the foreign plasmid was silenced after integration into the chromosome arm, whereas some IR/MAR plasmid repeats retained an open chromatin configuration, perhaps because the chromosomal integration site allowed it. Mtx treatment might select for cells bearing acetylated H3K9 on the plasmid, because DHFR protein should be expressed higher amount in these cells. Because the IR/MAR plasmid was integrated at multiple chromosomal locations within individual cells (see above), it should increase the likelihood that the plasmid might be inserted into a chromosomal environment that permits further amplification after Mtx treatment.

## Discussion

**Figure 8:**
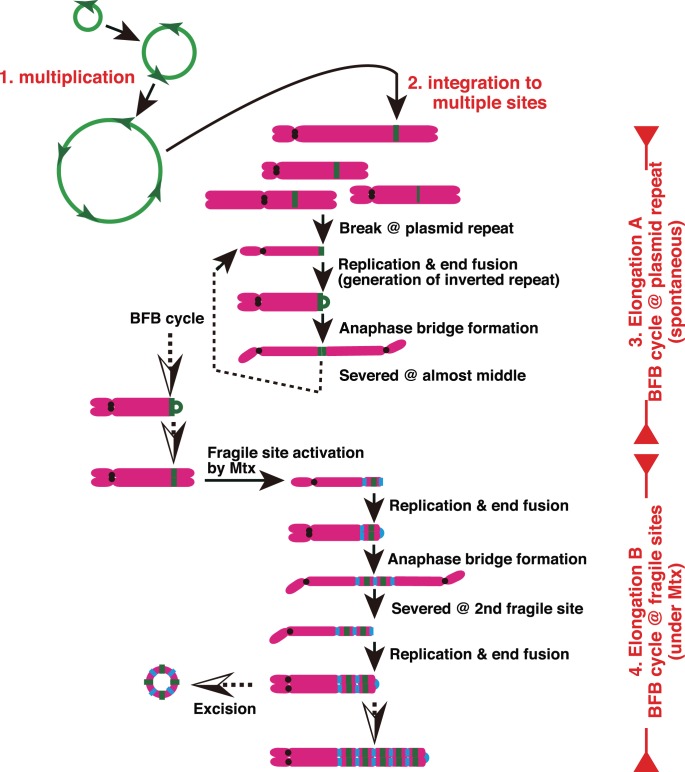
A model explaining how a circular molecule bearing the genomic initiation region/matrix attachment region (IR/MAR) sequence is amplified under replication stress. For a detailed explanation, see the Discussion section. Green: IR/MAR sequence; orange: chromosome arm; black circle: centromere; blue chromosome fragile site.

We have addressed how the IR/MAR sequence might accelerate the classical *Dhfr*/Mtx amplification, and why it might generate stable structure with high gene expression. Based on our result, we generated a model (Fig. 8), and our findings are discussed from this perspective.

The IR/MAR plasmid was multimerized before chromosomal integration (Fig. 8, step 1), consistent with the results of our previous study [6], [9], [10]. Furthermore, we now found that the multimer was integrated at many chromosomal sites (Fig. 8, step 2), increasing the probability that the plasmid multimer might be integrated into a “good” chromosomal site that enables further amplification and/or a higher level of gene expression. Therefore, the IR/MAR sequence might increase both the apparent HSR elongation rate and the level of gene expression.

At the chromosomal integration site, our results suggested the existence of two distinct BFB cycles: one operating at the plasmid repeat, and the other at the chromosomal fragile site. We previously characterized the former BFB cycle [9]. Whereas, the latter BFB cycle operating in the presence of Mtx was consistent to the published mechanism [17]–[19]. These two mechanisms work in turn to generate fine-ladder HSRs, and we refer to these steps as elongation A and B (Fig. 8, steps 3 and 4).

During elongation A, the BFB cycle proceeds at the plasmid repeat. This step occurs after integration of the plasmid sequence, regardless of whether it contains the IR/MAR sequence (Fig. 6). During this step, the anaphase bridge is always severed at the middle where the plasmid is located (Fig. 5C). Previously, we suggested the involvement of a cruciform structure, in the middle of a bridge, that determines the severing point [9]. Because the size of the cruciform might change, the severing point might be shifted relative to the exact middle, contributing to unequal segregation of HSRs to daughter cells and the elongation of HSRs in COLO 320 cells. HSRs produced by this mechanism are long homogenous arrays of plasmid repeats containing inverted repeats. In CHO DG44 cells, however, this mechanism did not contribute to HSR elongation, possibly because the cruciforms in these cells are small.

The BFB cycle that operates at the fragile site (elongation B) is a widespread mechanism of gene amplification. Mtx causes a nucleotide-deficient state by inhibiting DHFR enzyme activity, thereby stalling the replication fork; thus Mtx treatment results in the accumulation of double-strand breaks [15] and the activation of fragile sites [20], which, in turn, trigger the BFB cycle [21], [22]. For amplification to occur, two fragile sites are required (Fig. 8), consistent with an earlier report that two fragile sites define the boundary of the amplicon [17].

During each cycle of elongation A and B, an inverted repeat is generated within the amplified structure. Many remnants of this process might be detected in human cancer genomes [23]. A recent molecular analysis of the inverted repeat sequences in HSRs revealed how sister-chromatid fusion might occur: specifically, homology-mediated fold-back capping of broken ends followed by DNA replication is an underlying mechanism of sister-chromatid fusion [24]–[26]. Our analysis of the inverted repeats in HSRs generated from the IR/MAR plasmid in COLO 320 cells also reached the same conclusion [13].

Both elongation A and B are likely to depend on the chromosomal site where the plasmid is located; indeed, the efficiency of gene amplification depends heavily on the chromosomal site (Fig. 3). Therefore, in this study the conventional plasmid lacking the IR/MAR was hardly amplified, because it was usually integrated into only one chromosomal site. By contrast, the IR/MAR plasmid was integrated to multiple chromosomal sites, dramatically increasing the probability of gene amplification.

After growth in the presence of Mtx, the level of histone H3K9 acetylation in the plasmid sequence increased, whereas H3K9 methylation decreased. Mtx lowers the concentration of S-adenosyl methionine, thereby decreasing global levels of DNA methylation [27]; the drug might have a similar effect on histone methylation. In addition, Mtx can inhibit the histone deacetylase complex, albeit at a rather high effective dose [28], and such direct activities of Mtx might increase H3K9 acetylation. In addition, our data suggested that cells bearing the plasmid in open chromatin might survive in the presence of Mtx, because clones with acetylated H3K9 elongated their HSRs, whereas the clone with methylated H3K9 died (Fig. 7). This all demonstrates that that chromosomal recombination is active in acetylated chromatin and inactive in methylated chromatin, e.g., H3K9 methylation is related to the stability of rDNAs and satellite repeats [29], and acetylated H3K9 is related to hotspots for meiotic recombination [30]. Therefore, an HSR with acetylated H3K9 might be excised to generate a ring chromosome (Fig. 6B and also depicted in Fig. 8). Such an effect would be accelerated in the presence of Mtx, because Mtx is a clastogenic agent, and this mechanism could explain why the amplified structures generated by Mtx were usually unstable. The involvement of the IR/MAR sequence generates stable structures even in the absence of Mtx, and the amplicon generated by IR/MAR remained open if it was placed in a good chromosomal location (Fig. 7E).

The model proposed here (Fig. 8) is a fusion of the episome model and the BFB-cycle model. The former model [31] involves a submicroscopic episome that mediates gene amplification. Such episomes have been detected during amplification of a multi-drug-resistance gene [32] and of the breast cancer-related gene PIP [33]. Furthermore, the *MYC* gene amplification detected in primary tumor cells is mediated by extrachromosomal amplification followed by chromosomal integration [34], [35]; thus the IR/MAR plasmid appears to mimic the episomal model of gene amplification. On the other hand, circular molecules excised from the chromosome arm have been observed as extrachromosomal closed circular (ecc) DNAs [36]–[38] or small polydisperse circular (spc) DNAs [39]. Furthermore, recent techniques have identified tens of thousands of kinds of extrachromosomal circular molecules (micro DNAs) in cells from normal mouse tissues or human cell lines [40]. Because IR/MAR sequences are scattered throughout the human genome at 50–100 kb intervals, it is very likely that some circular molecules of genomic origin contain IR/MARs, and therefore behave similarly to the IR/MAR plasmid.

The IR/MAR plasmid was amplified to form a direct repeat during extrachromosomal amplification, followed by amplification on the chromosome arm by the BFB cycle that generates inverted repeats (discussed above). By contrast, direct repeat amplification has been observed in cancer cells taken directly from patients, e.g., in esophageal adenocarcinoma bearing *ERBB2* amplifications [41], and in gastric carcinoma bearing amplification of the 17q21 locus, which is not linked to common fragile sites [42]. rDNA repeats are also direct repeats. In general, direct repeats can be generated far more easily than inverted repeats, because the former structure can be generated by homologous recombination. Taken together, our data indicate that under replication stress such as Mtx, amplification of a circular molecule bearing an IR/MAR of genomic origin is a natural process. The resultant fine-ladder HSRs are similar to those detected in primary cancers and thus the model we propose on the basis of our findings (Fig. 8) might plausibly explain the gene amplifications detected in a wide range of human cancers.

## Supporting Information

Table S1 PCR primers used in this study are listed.
(DOC)
