# Supplementary material for: How a Replication Origin and Matrix Attachment Region Accelerate Gene Amplification under Replication Stress in Mammalian Cells
Source: PLoS One. 2014 Jul 25;9(7):e103439. doi: 10.1371/journal.pone.0103439 (PMC4111587; doi:10.1371/journal.pone.0103439)
Supplement: Table S1 — PCR primers used in this study are listed. (DOC) [file pone.0103439.s001.doc]

Table S1

Primers

| No. | Name | Sequence |
| --- | --- | --- |
| 1 | SRalfa 487L | GGTGCCTCCTGAACTACGTC |
| 2 | SFV 1519R | CTGTAGGCATAGGCTTGGTT |
| 3 | DHFR 4.6K-5' | CCGCAAGCTTTAGAAATTCA |
| 4 | DHFR IR 4621L | GGGGCGCGCCCAGCAATAGAAACCCCAGCT A |
| 5 | SFV dhfr 6131R | TCTGAGACTTGGCTGGGAAG |
| 6 | Col E1 8752L | GTCCAGGCAGGTAGATGACG |
| 7 | Am 10014L | ATACGGGAGGGCTTACCATC |
| 8 | SRalfa 497R | CTAGACGGCGGACGTAGTTC |
| 9 | BS 1303R | GAAATCAACTCCCTACACATA |
| 10 | DHFR 1817R | GAGAAATCAAAGGCCCACCT |
| 11 | DHFR IR 2811R | GGGGCGCGCCCAAGAATATTCATTATA AAAGGCCCTA |
| 12 | DHFR IR 4021L | GGGGCGCGCCGAGCTCCAATGCCCTCTTTT |
| 13 | DHFR IR 4961L | GGGGCGCGCCTTCAGCCTTAACCTTTGTTCTGT |
| 14 | DHFR 4.6K-3' | GAATCCATTGTGTGGCTCCT |
| 15 | Col E1 8752R | CGTCATCTACCTGCCTGGAC |
| 16 | Am 10510R | CGCCGCATACACTATTCTCA |

Set of primers used

| Primer set No. | Name | Name |
| --- | --- | --- |
| 1 | SRalfa 487L | SFV 1519R |
| 2 | SFV 1519R | DHFR 4.6K-5' |
| 3 | DHFR 4.6K-5' | DHFR IR 4621L |
| 4 | DHFR IR 4621L | SFV dhfr 6131R |
| 5 | SFV dhfr 6131R | Col E1 8752L |
| 6 | Col E1 8752L | Am 10014L |
| 7 | Am 10014L | SRalfa 487L |
| 8 | SRalfa 497R | BS 1303R |
| 9 | BS 1303R | DHFR 1817R |
| 10 | DHFR 1817R | DHFR IR 2811R |
| 11 | DHFR IR 2811R | DHFR IR 4021L |
| 12 | DHFR IR 4021L | DHFR IR 4961L |
| 13 | DHFR IR 4961L | DHFR 4.6K-3' |
| 14 | DHFR 4.6K-3' | Col E1 8752R |
| 15 | Col E1 8752R | Am 10510R |
| 16 | Am 10510R | SRalfa 497R |
| 17 | SFV 1519R | Col E1 8752L |
| 18 | Col E1 8752L | Am 10014L |
| 19 | BS 1303R | Col E1 8752R |
| 20 | Col E1 8752R | Am 10510R |
